# Increased expression of KPNA2 predicts unfavorable prognosis in ovarian cancer patients, possibly by targeting KIF4A signaling

**DOI:** 10.1186/s13048-021-00818-9

**Published:** 2021-05-25

**Authors:** Xiangrong Cui, Honghong Wang, Xueqing Wu, Kai Huo, Xuan Jing

**Affiliations:** 1 Reproductive Medicine Center, Children’s Hospital of Shanxi and Women Health Center of Shanxi, Affiliated of Shanxi Medical University, Taiyuan, 030001 China; 2 Gynaecology and Obstetrics Department, Children’s Hospital of Shanxi and Women Health Center of Shanxi, Affiliated of Shanxi Medical University, Taiyuan, 030001 China; 3 Breast Surgery Department, Tumor Hospital of Shanxi, Affiliated of Shanxi Medical University, Taiyuan, 030000 China; 4 Clinical Laboratory, Shanxi Prov. People’s Hospital, Affiliated of Shanxi Medical University, Taiyuan, 030001 China

**Keywords:** KPNA2, Ovarian cancer, Prognosis, KIF4A

## Abstract

**Background:**

Karyopherin α-2 (KPNA2) is a member of karyopherin family, which is proved to be responsible for the import or export of cargo proteins. Studies have determined that KPNA2 is associated with the development and prognosis of various cancers, yet the role of KPNA2 in ovarian carcinoma and its potential molecular mechanisms remains unclear.

**Materials and methods:**

The expression and prognosis of KPNA2 in ovarian cancer was investigated using GEPIA and Oncomine analyses. Mutations of KPNA2 in ovarian cancer were analyzed by cBioPortal database. The prognostic value of KPNA2 expression was evaluated by our own ovarian carcinoma samples using RT-qPCR. Subsequently, the cell growth, migration and invasion of ovarian cancer cells were investigated by CCK-8 and transwell assay, respectively. The protein levels of KPNA2 and KIF4A were determined by western blot.

**Results:**

We obtained the following important results. (1) KPNA2 and KIF4A wereoverexpressed in ovairan cancer tissues and cells. (2) Among patients with ovarian cancer, overexpressed KPNA2 was associated with lower survival rate. (3) Mutations (R197* and S140F) in KPNA2 will have some influences on protein structure, and then may cause protein function abnormal. (4) KPNA2 konckdown inhibited proliferation, migration, invasion, as well as the expression of KIF4A.

**Conclusion:**

KPNA2, as a tumorigenic gene in ovarian cancer, accelerated tumor progression by up-regulating KIF4A, suggesting that KPNA2 might be a hopeful indicator of treatment and poor prognosis.

## Introduction

Ovarian cancer (OC), a common gynecological malignancy, is the fifth leading cause of death among women around the world [10, 28]. According to statistics published in National comprehensive Cancer Network (NCCN), more than half of patients can not live more than 5 years after diagnosis [1]. A number of genetic alterations associated with development and progression of ovarian cancer have been reported; however, most patients are in the advanced stage at the time of initial diagnosis [13, 21, 31]. It is urgent to reveal the pathophysiological mechanism of ovarian cancer, optimize the treatment plan, and realize the early diagnosis of ovarian.

Karyopherin α-2 (KPNA2) is a member of karyopherin α family (KPNA), which plays an important role in nucleocytoplasmic transport [3, 18, 29]. Studies have determined that KPNA2 is associated with modulating both the nuclear import and the cytoplasm export of molecules [4, 9, 20]. Recently, higher expression of KPNA2 was demonstrated in breast cancer [8], burkitt lymphoma [23], melanoma [33], bladder cancer [25], and correlated with the poor prognosis of patients. However, the exact role of KPNA2 in ovarian cancer and its underlying molecular mechanism have not been elucidated.

In current research, we hypothesized that KPNA2 may be a promising candidate for the potential diagnostic and prognosticof ovarian cancer. To confirm this hypothesis, we used bioinformatics methods to analyze the expression and prognostic value of KPNA2 in ovarian cancer. Furthermore, we used ovarian cancer cell lines to identify the molecular mechanisms underlying the effect of overpressed KPNA2 on ovarian carcinoma. Our results determined that KPNA2 is overexpressed in ovarian carcinoma and plays an important role in malignant transformation through regulating KIF4A signaling pathway. These observational findingswill contribute to the development and optimization of novel insights into diagnosis and prognosis for ovarian carcinoma.

## Materials and methods

### Clinical samples

This study was performed on 35 patients with ovarian carcinoma from Shanxi province of China. Frozen tissues were collected from the Department of Gynaecology and Obstetrics from Tumor Hospital of Shanxi (China). The average age of the patients was 60.14 years (23–77 years). The study was performed following the Declaration of Helsinki set of principles and approved by the Ethics Committee of the Shanxi Medical University (Ethical code: 201922021). Informed written consent was obtained from all patients enrolled.

### Cell culture and transfections

The human ovarian carcinoma SKOV3 cells were purchased from American Type Culture Collection. Cells were cultured in DMEM/F12 medium supplemented with 10% FBS (Gibco, Thermo Fisher Scientific, Inc.) in a humidified atmosphere with 5% CO_2_ at 37 °C. siRNA targeting KPNA2 (si-KPNA2) and siRNA negative control (si-NC) were constructed and purchased from GenePharma (Shanghai, China). Cell transfection was performed using the Lipofectamine 20,000 (Invitrogen, Carlsbad, CA, USA) according to the manufacturer’s instructions for 48 h.

### Gene expression profiling interactive analysis

Gene Expression Profiling Interactive Analysis (GEPIA) database (http://gepia.cancer-pku.cn/) was utilized to analyze the RNA sequencing expression of 9736 tumors and 8587 normal samples from The Cancer Genome Atlas (TCGA) and Genotype-Tissue Expression (GTEx) project data [26]. In GEPIA, KPNA2 expression various human cancers and adjacent normal tissues was obtain, furthermore the KPNA2 expression in ovarian carcinoma and corresponding normal tissues was validated.

### Oncomine database analysis

KPNA2 gene expression levels in malignancies were analyzed from Oncomine database, a network based data mining platform including 715 datasets and 86,733 samples (http://www.oncomine.org) [24]. Paired Student’s t-test was performed to compare group means. A fold-change of at least 2 with a *P*-value < 0.0001 was defined as clinically significant, as previously described.

### cBioportal database analysis

The term “KPNA2” was adopt to search the cBio Cancer Genomics Portal (cBioPortal) database (http://www.cbioportal.org/) and ovarian serous cystadenocarcinoma (TCGA, Nature 2011, *n* = 489) cohort was performed [6, 11]. Mutations, putative copy number aberrations, and co-expression from RNA-seq data, were assessed as the search parameters to visualize the results.

### University of California Santa Cruz Cancer Genomics Browser Analysis

The University of California Santa Cruz (UCSC) Xena browser (http://xena.ucsc.edu/), included 758 cases of ovarian cancer with genomic and clinical, was performed to access TCGA ovarian cancer data. The relationship between the transcriptional expression of KPNA2 and KIF4 was accessed through ovarian carcinoma cohort in TCGA database (TCGA-Ovarian Cancer) [12].

### UALCAN analysis

UALCAN (http://ualcan.path.uab.edu), an interactive web resource, is performed to analyze relative mRNA expression of potential genes (TCGA and MET500 transcriptome sequencing) and relationship of the mRNA expression with various tumor subtypes, including age, gender, tumor stages, and other clinicopathological features [7]. In our research, UALCAN was utilized to access the mRNA expression of KPNA2 in primary ovarian carcinoma tissues and its association with various tumor molecular subtypes and clinicopathologic parameters.

### PrognoScan online platform

PrognoScan online platform (http://www.prognoscan.org) was utilized to access the prognostic value of KPNA2 expression in ovarian carcinoma patients [19]. In this web based online platform, the *P*-value, HR and 95% confidence intervals (CI) based on a KPNA2 expression was automatically calculated by PrognoScan.

### Kaplan-Meier plotter analysis

The Kaplan Meier plotter (http://kmplot.com/analysis/) is capable to evaluate the role of 54 k genes on prognosis in various cancer types from the databases containing GEO, EGA, and TCGA. We accessed the association between the KPNA2 expression and prognosis using the ovarian cancer cohort [22].

### CCK-8 detection of viability

Cell Counting Kit-8 (CCK-8, ATgene, Taiwan, China) was performed to evaluate the cell viability. Cells were tiled into 96-well plates at 5 × 10^3^ cells per well and subjected in 100 μl serum-free medium for 24, 48, 72, and 96 h. Then, 10% CCK-8 solution was added to each well for 2 h and the absorbance (A) valuewas detected at 450 nm by microplate reader (BioTek, Epoch, VT). All tests were repeated eight times, and each experiment was carried out at least three replicate.

### Cell migration and invasion ability assay

Invasion and migration abilities of ovarian cancer cells were determined using Boyden assay. 600 μl DMEM/F12 supplemented with 10% FBS was added to the 24-well plate, followed by Transwell chamber (Millipore, Burlington, MA, USA) seeded with 4 × 10^4^ cells. For cell invasion assay, 100 μg/ml Matrigel (BD Biosciences, Boston, MA, USA) was added to the upper layer of chamber. After incubation for 24 h at 37 °C, the cells in the upper chamber were removed. Cells were fixed with 600 μl fixative solution for 10 min. The chamber was stained with 600 μl crystal violet for 15 min. Finally, an inverted microscope (Eclipse Ti2; Nikon Corporation) was used to photograph and calculate the invading and migratory cells.

### Quantitative real-time PCR (qRT-PCR)

RNA extraction kit was performed to extract the total RNAs from tissues and cells. RNA was reverse transcribed into complementary DNA by Prime Script™ RT Master Mix kit. qRT-PCR was performed using SYBR® Premix Ex Taq Kit. For PCR amplification, GAPDH was presented as an internal reference. The primer sequences were as follows: KPNA2, 5′-ATTGCAGGTGATGGCTCAGT-3′ (forward) and 5′-CTGCTCAACAGCATCTATCG-3′ (reverse); GAPDH, 5′-ACCACAGTCCATGCCATCAC-3′ (forward); and 5′-TCCACCA CCCTGTTGCTGTA-3′ (reverse). The method of 2^−ΔΔCt^ was performed to calculate the relative gene expression.

### Western blot analysis

Proteins from ovarian carcinoma cell lines were extracted with lysis buffer follow instructions. The BCA method was performed to determine protein concentration using BCA Protein Assay kit. Protein lysate was subjected to SDS-PAGE gels and transferred subsequently onto PVDF membranes. The membrane was blocked by TBS containing 0.1% Tween-20. Then, membranes were incubated with rabbit anti KPNA2 monoclonal antibody (ab170495; 1:1000; Abcam, Cambridge, MA, USA), KIF4A (ab124903; 1:1000; Abcam, Cambridge, MA, USA) or GAPDH (cat. no. T0004; 1:5000; Affinity Biosciences) at 4 °C overnight, followed by goat anti-rabbit IgG-HRP secondary antibodies for 2 h at 37 °C. ECL reagents were performed to visualize protein bands.

### Statistical analysis

GraphPad Prism 7.0 was performed to statistical analysis. Student’s t-test was adopt to analysis differences between two groups. Overall survival was calculated by Kaplan-Meier survival curves and the statistical comparisons were analyzed by Log-rank test. *P*-value < 0.05 is applied as the threshold.

## Results

### KPNA2 transcript expression status in human ovarian Cancer

**Fig. 1:**
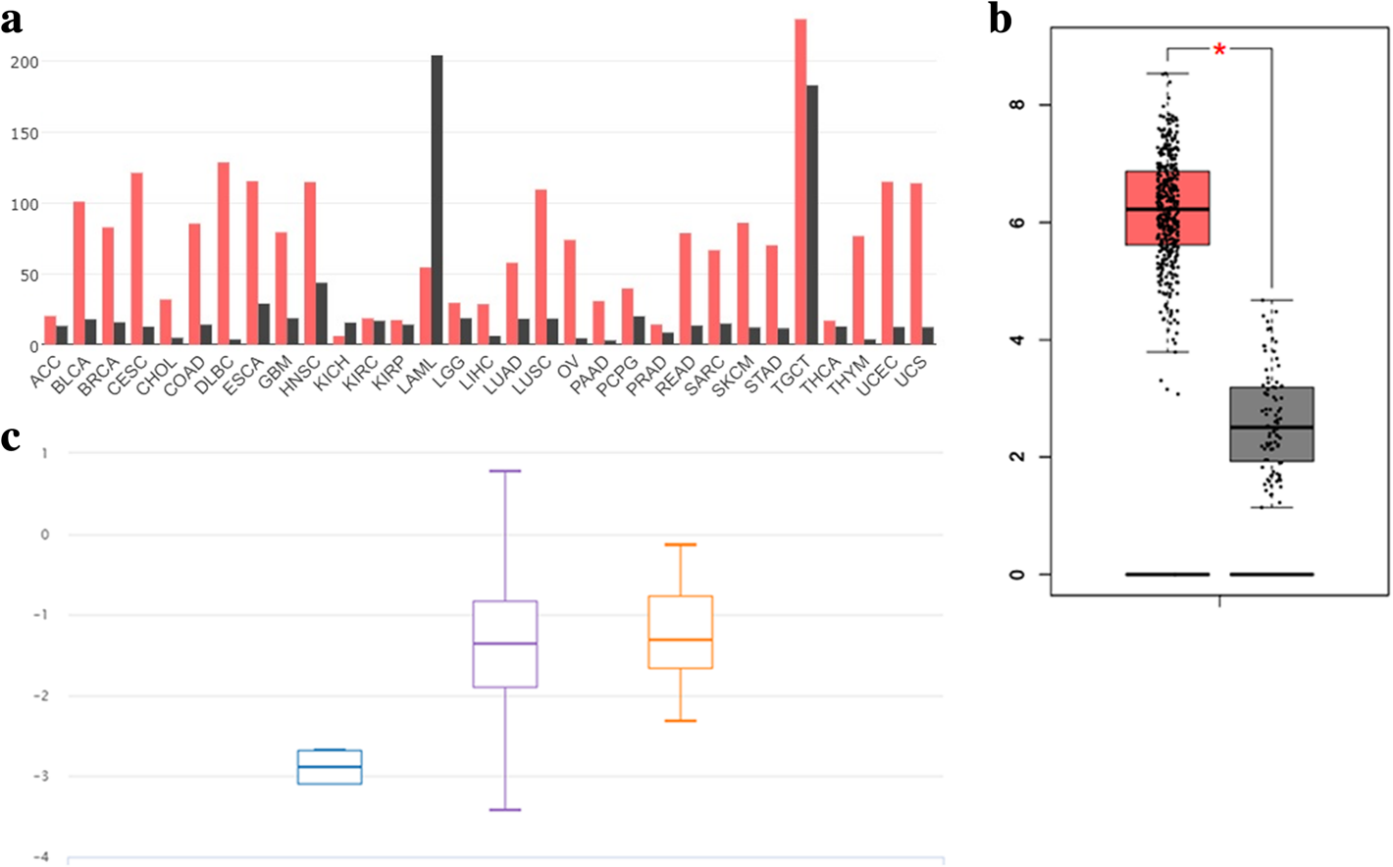
Expression of KPNA2 in ovarian cancer and normal tissues from online databases. (**a**) KPNA2 expression profile across all tumor samples (red) and paired normal tissues (black) from GEPIA. (**b**) The expression of KPNA2 mRNA in ovarian cancer tissues (red box) and paried normal tissues (black box) from GEPIA. (**c**) Expression of KPNA2 was higher in primary and recurrent ovarian cancer compared with normal ovarian tissues from UCSC Xena browser

**Fig. 2:**
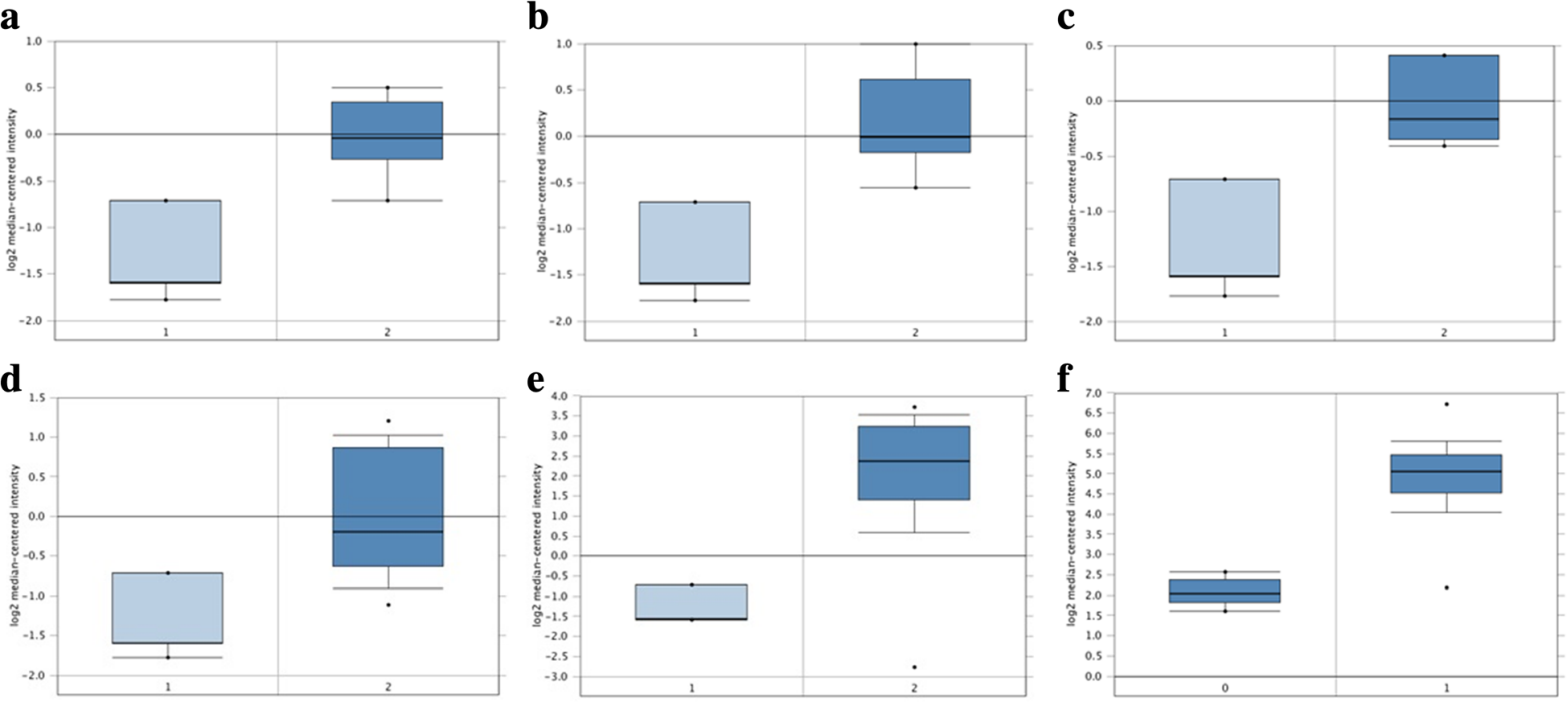
KPNA2 analysis in ovarian cancer (Oncomine database). The box plot compares KPNA2 expression in cancer samples (right) and matches normal (left) samples generated from the Oncomine database. (**a**) ovarian mucinous adenocarcinoma; (**b**) ovarian endometrioid adenocarcinoma; (**c**) ovarian clear cell adenocarcinoma; (**d**) ovarian serous adenocarcinoma; (**e**) ovarian serous surface papillary carcinoma, (**f**) ovarian serous cystadenocarcinoma

The expression profile of *KPNA2* was identified by GEPIA. The data revealed that *KPNA2* was significantly higher in BLCA, BRCA, CESC, COAD, DLBC, ESCA, GBM, HNSC, LIHC, LUAD, LUSC, OV, PAAD, READ, SKCM, STAD, THYM, UCEC, UCS (Fig. 1a and b). To further confirm this result, the UCSC Xena browser was carried out to determine the expression profile of KPNA2, which was higher expression in primary and recurrent ovarian carcinoma compared with normal ovarian tissues (Fig. 1c). Then, the Oncomine database was adopted to evaluate KPNA2 expression in different histologic subtypes of primary ovarian cancers. KPNA2 expression was significantly higher in ovarian mucinous adenocarcinoma, ovarian endometrioid adenocarcinoma, ovarian clear cell adenocarcinoma, ovarian serous adenocarcinoma, ovarian serous surface papillary carcinoma, ovarian serous cystadenocarcinoma (Fig. 2 and Table 1).

**Table 1:** KPNA2 expression in ovarian cancer

Cancer subtype	***p***-value	Fold change	***t***-test	Rank (%)	Sample	Reference
Ovarian Mucinous Adenocarcinoma	2.02E-4	2.523	5.742	1	9	8
Ovarian Endometrioid Adenocarcinoma	6.60E-5	2.891	6.067	1	9	8
Ovarian Clear Cell Adenocarcinoma	2.30E-4	2.554	5.987	1	7	8
Ovarian Serous Adenocarcinoma	1.07E-4	2.544	5.330	4	20	8
Ovarian Serous Surface Papillary Carcinoma	1.57E-8	11.343	10.339	2	28	8
Ovarian Serous Cystadenocarcinoma	3.72E-9	7.566	24.875	1	586	TCGA (No Associated Paper)

### Genetic alterations in KPNA2 and Clinicopathological parameters

**Fig. 3:**
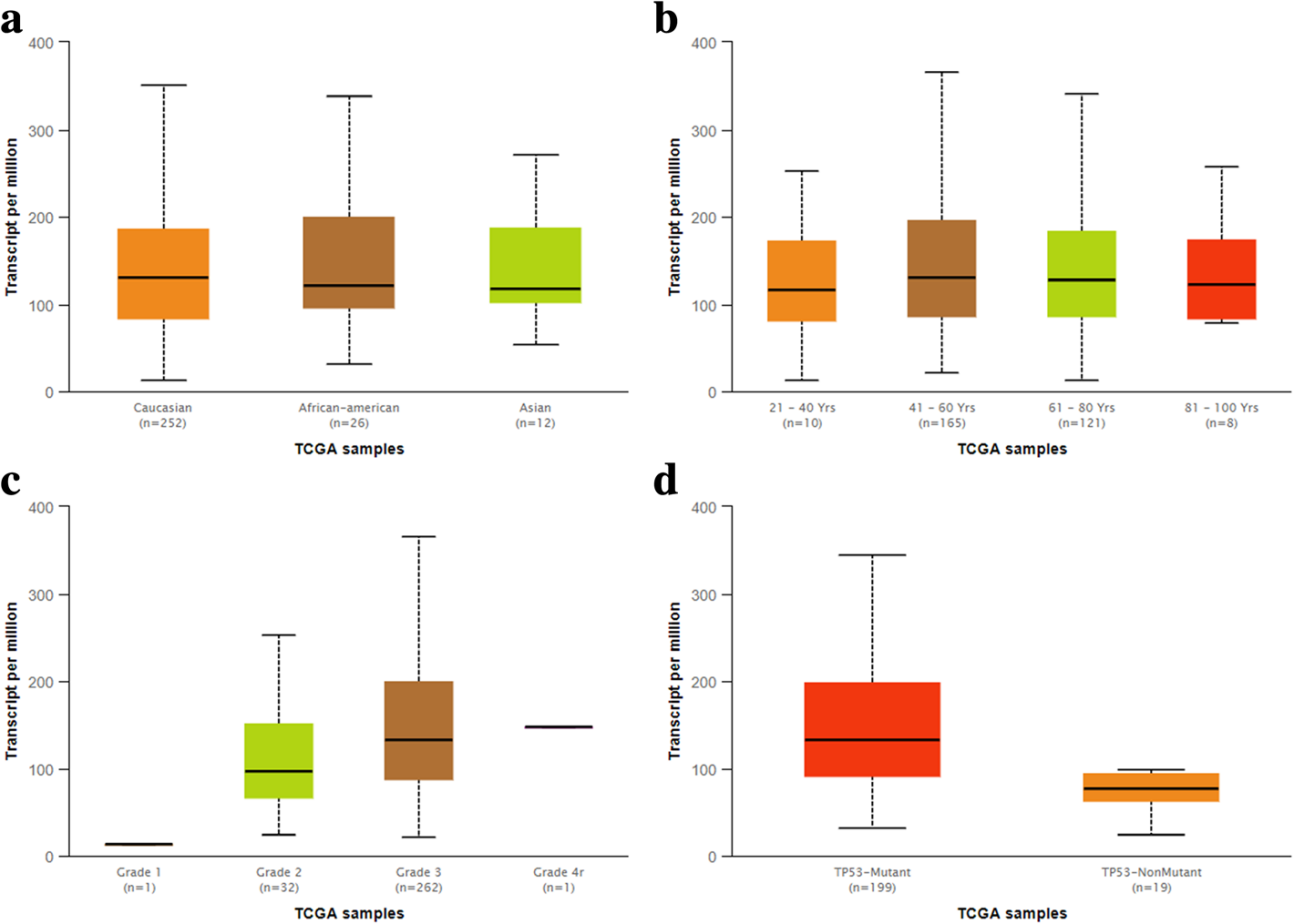
Genetic alterations of KPNA2 in ovarian cancer subgroups. Subgroup analysis of multiple clinic pathologic features of ovarian cancer samples in the TCGA. (**a**) rases; (**b**) ages; (**c**) stages; (**d**) TP53-Mutant

Further subgroup analysis according multiple clinic pathologic features of ovarian carcinoma in the TCGA was shown in Fig. 3. The results determined that KPNA2 expression level was not significantly different among different races and age groups (Fig. 3a and b). In addition, the expression level of KPNA2 was significantly associated with the stages of ovarian cancer (Fig. 3c). A significant overexpression of KPNA2 in TP53-Mutant samples than TP53-NonMutant samples of ovarian cancer in subgroup analyses based on whether TP53 is mutated (Fig. 3d).

### KPNA2 mutations in ovarian Cancer

**Fig. 4:**
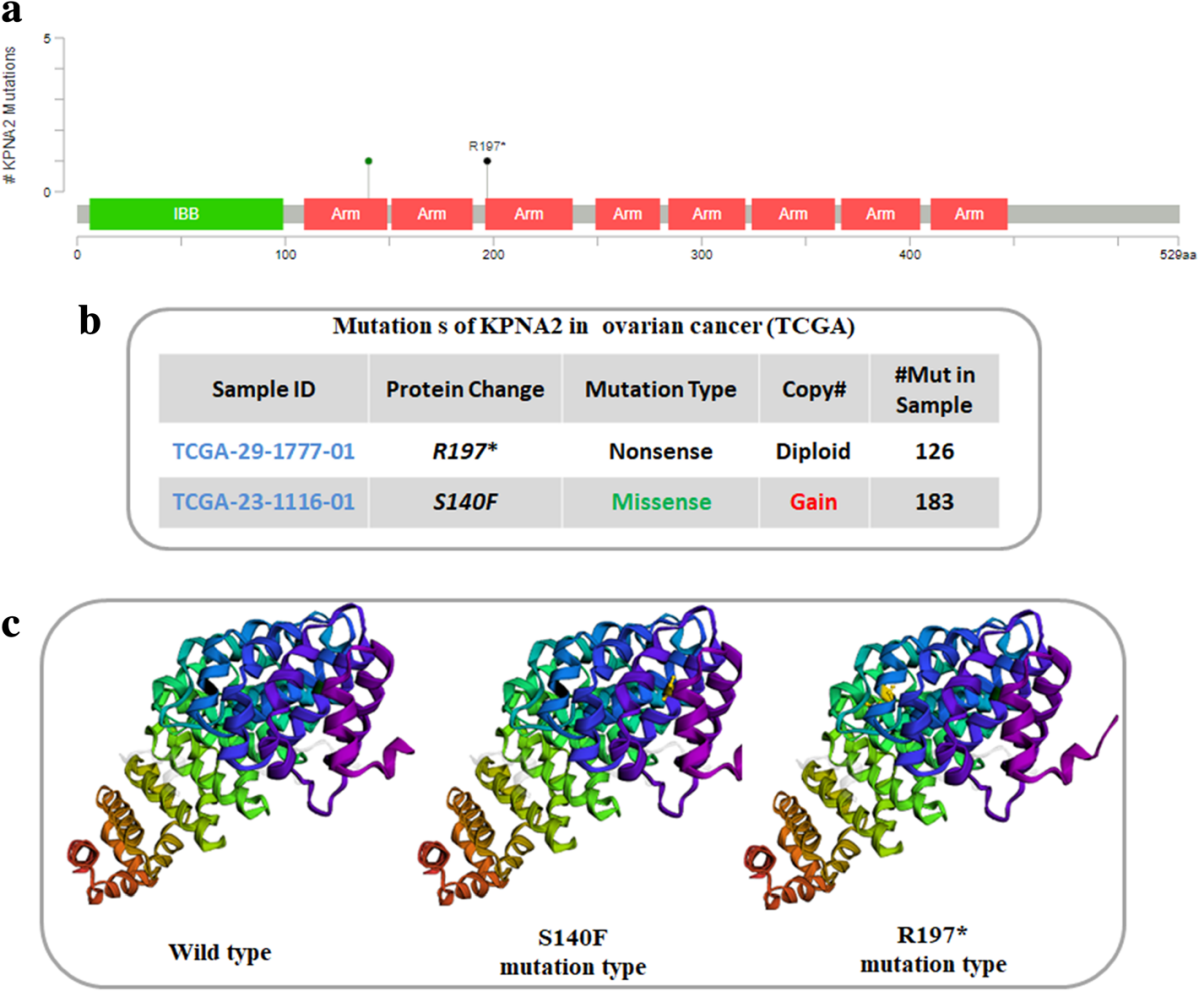
KPNA2 Mutations in Ovarian Cancer. (**a**) The graphical view shows the Pfam protein domains and the positions of specific mutations. The length of the line connecting the mutation annotation to the protein is indicative of the number of samples that have the mutation. The most recurrent mutations are labeled in the graphical view. (**b**) The tabular view provides additional information about all mutations in each query gene. (**c**) 3D protein structure visualization and analysis were performed

Mutations in ovarian cancer mainly occur in Arm domain, which analyzed by cBioPortal database. As shown in Fig. 4, the graphical view reveals the KPNA2 protein domains and the positions of specific mutations. The most recurrent mutations are labeled in the graphical view (Fig. 4a). Furthermore, the additional information about all mutations (R197* and *S140F*) in KPNA2 was revealed in tabular view (Fig. 4b). 3D protein structure visualization and analysis were performed using cBioPortal database. As shown in the Fig. 4c, Ser140 was replaced by Phe, while the R197 was replaced by termination codon. Therefore, the R197* and *S140F* mutations will have some influences on protein structure, and then may cause protein function abnormal.

### Relationship of KPNA2 expression and prognosis in ovarian cancer

**Fig. 5:**
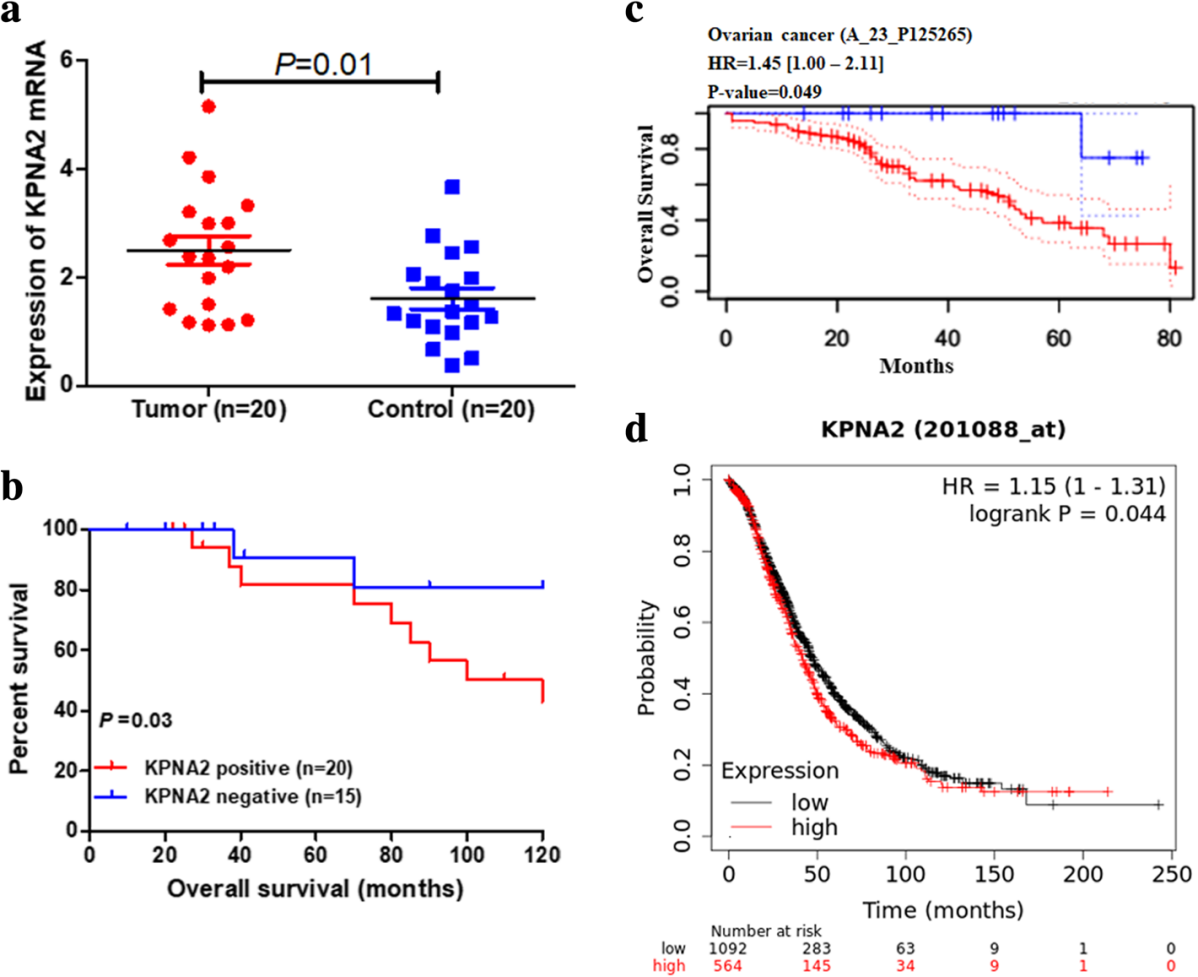
KPNA2 as a prognosis marker in ovarian cancer. (**a**) Expression of KPNA2 in tumor (20 cases) and adjacent normal tissues (20 cases). (**b**) Kaplan-Meier curves based on KPNA2 expression were drawn for overall survival in 35 patients. (**c**) Overall survival (OS) curves calculated by PrognoScan tool. (**d**) OS curves calculated by Kaplan-Meier Plotter Analysis

To evaluate whether KPNA2 expression level has predictive significance for ovarian carcinoma prognosis, we detected the expression of KPNA2 mRNA and its relationship with prognosis. Our own results found the upregulation of KPNA2 mRNA expression and worse probabilities of survival in ovarian carcinoma (Fig. 5a and b). Furthermore, we used PrognoScan Online Platform and Kaplan-Meier Plotter Analysis to further confirm the predictive value of KPNA2 expression for ovarian prognosis. Both databases verified the KPNA2 expression was negatively related to overall survival (PrognoScan Online Platform: HR = 1.45 [1.00–2.11], *p* = 0.049; Kaplan-Meier Plotter Analysis: HR = 1.15 [1.00–1.31], *p* = 0.044) (Fig. 5c and d).

### Co-expression of KPNA2 gene

**Fig. 6:**
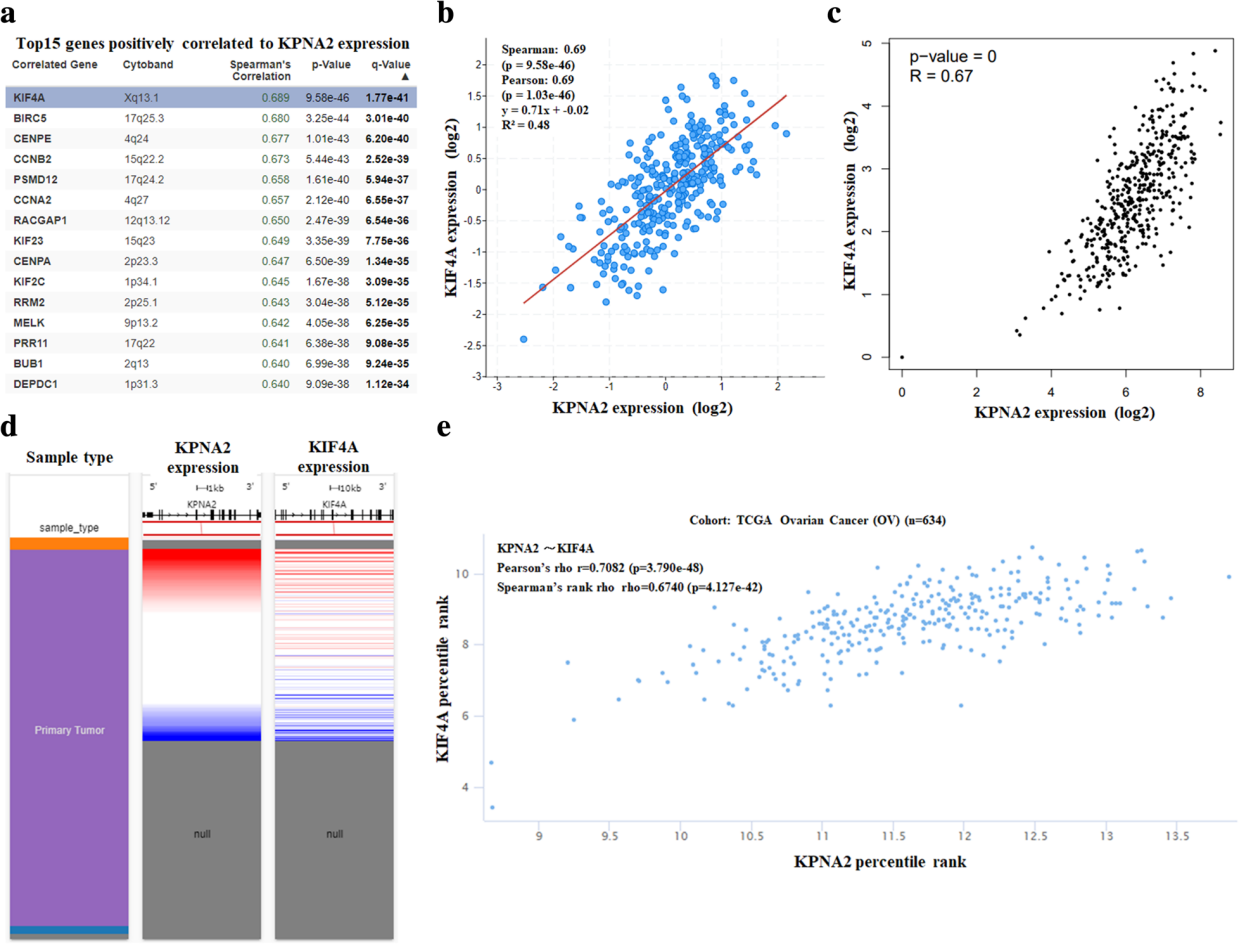
Co-Expression of KPNA2 gene. (**a**) Co-expression of KPNA2 gene as determined by cBioPortal. (**b**) Regression analysis between KPNA2 and KIF4A in ovarian performed by cBioPortal. (**c**) Relationship between KPNA2 and KIF4A in ovarian cancer through GEPIA. (**d**) Heat map of KPNA2 and KIF4A mRNA expression in ovarian cancer identified by UCSC Xena. (**e**) Correlation between KPNA2 and KIF4A mRNA expression in the TCGA database, identified by UCSC Xena

**Fig. 7:**
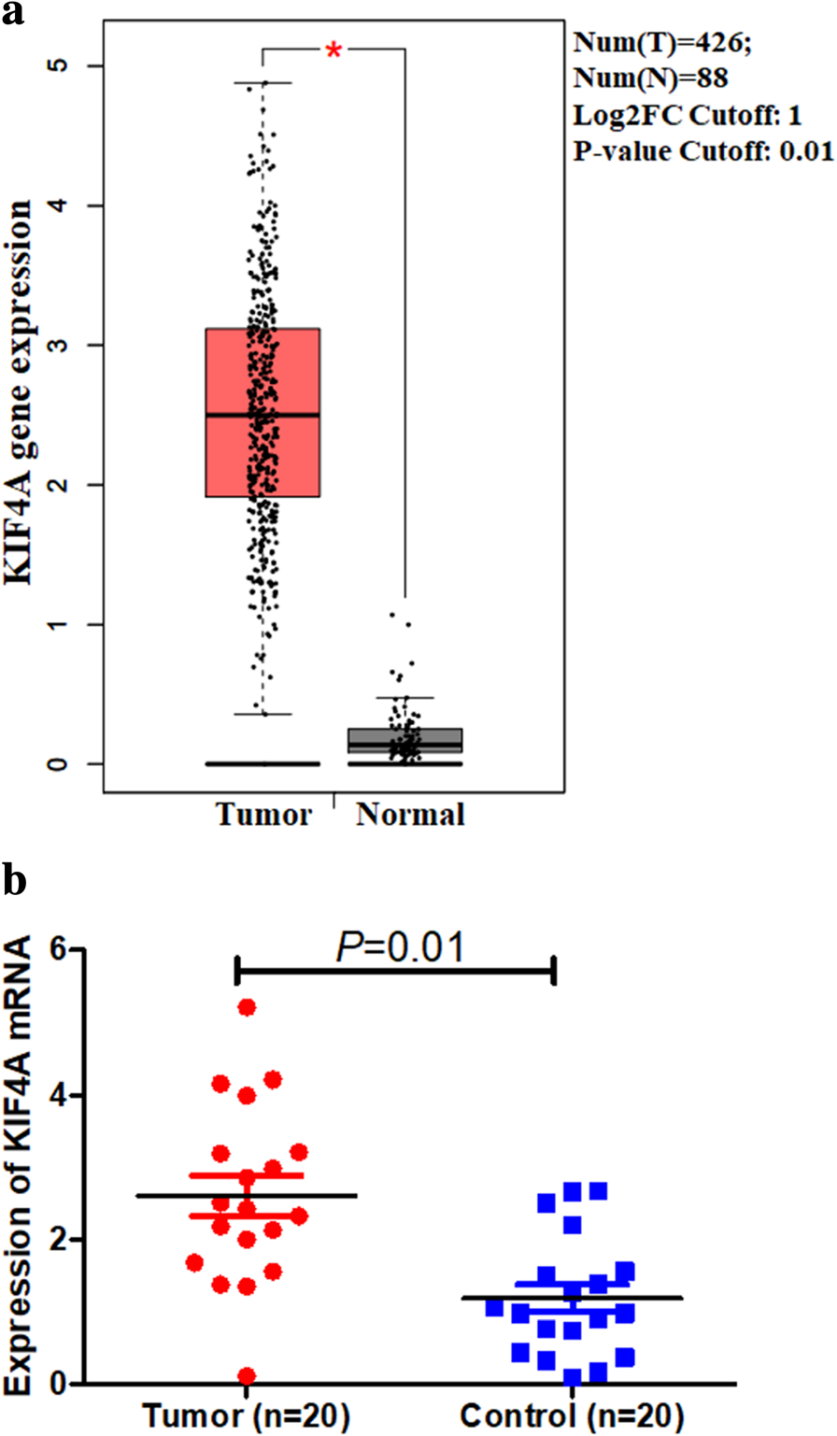
Expression of KIF4A in ovarian cancer. (**a**) The expression of KIF4A mRNA in ovarian cancer tissues (red box) and paired normal tissues (black box) from GEPIA. (**b**) Expression of KIF4A in tumor (20 cases) and adjacent normal tissues (20 cases)

To further determine the regulatory mechanisms underlying the role of *KPNA2* in ovarian carcinoma, data mining was performed on a ovarian carcinoma cohort using cBioPortal. Kinesin family member 4A (KIF4A) is a highly related gene (Fig. 6a); it promoted progression in various tumor tissues by regulating chromosome segregation machinery in mediating spindle organization and cytokinesis [2, 5, 32]. A regression analysis using cBioportal showed that *KPNA2* and *KIF4A* levels are highly correlated (Pearson’s correlation = 0.69; Spearman’s correlation = 0.69) (Fig. 6b). The positive correction between *KPNA2* and *KIF4A* mRNA expression was determined using data from GEPIA (Fig. 6c). By investigating ovarian carcinoma data in TCGA using UCSC Xena, the positive correlation was further confirmed (Fig. 6d and e). These data informed that *KPNA2* could be associated with the *KIF4A* pathway in ovarian carcinoma. To investigate the genetic alterations of *KIF4A*, the GEPI tool was applied to determine *KIF4A* expression profiles. The results of *KIF4A* analysis informed that *KIF4A* highly expressed in ovarian carcinoma tissues compared with normal ovarian tissues (Fig. 7a). Then, our own results further confirmed the overexpression of *KIF4A* mRNA and worse probabilities of survival in ovarian carcinoma (Fig. 7b).

### KPNA2 accelerated the biological characteristics of ovarian Cancer cells through up-regulating KIF4A

**Fig. 8:**
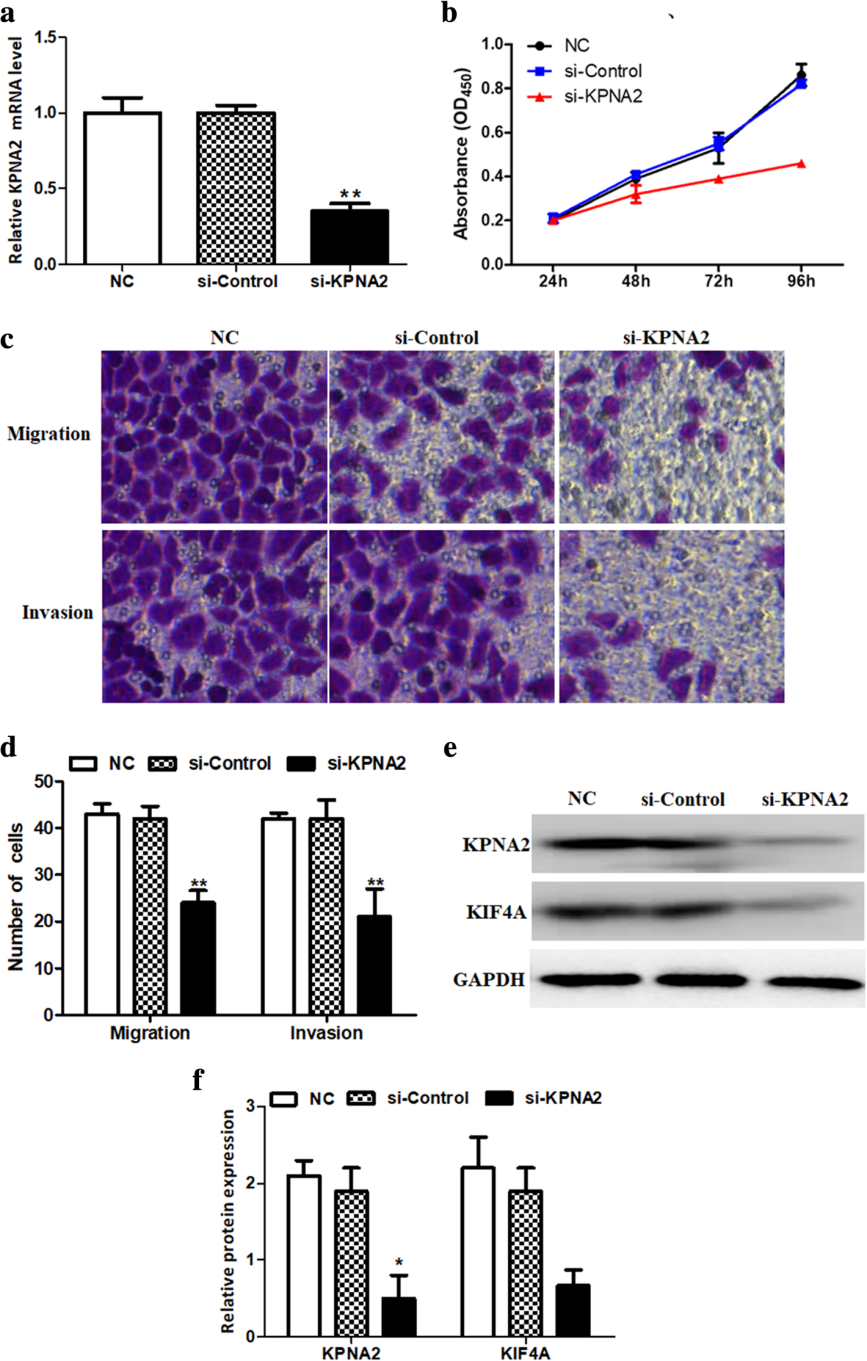
KPNA2 accelerated the cell growth, invasion, and migration in ovarian cancer through KIF4A. (**a**) The expression of KPNA2 was prominently reduced in ovarian cancer cells transfected with si-KPNA2. (**b**) The cell proliferation of ovarian cancer cell lines transfected with different interference sequences, cell proliferation was the weakest in si-KPNA2 group. (**c** and **d**) Invasion and migration assays of two kinds of cancer cells under different interference conditions. (**e** and **f**) The western blot showed KIF4A expression was significantly decreased in si-KPNA2 group ovarian cells. *P<0.05 and **P<0.01, compared with NC group

To access the potential role of KPNA2 in ovarian cancer cells, the endogenous expression level of KPNA2 was knocked down with si-KPNA2 transfection. As exhibited in Fig. 8a, the KPNA2 expression was significantly reduced in ovarian cancer cells transfected with si-KPNA2. Next, CCK-8 assay demonstrated that KPNA2 knockdown significantly weakened the ability of proliferation of ovarian carcinoma cells (Fig. 8b). Furthermore, transwell assay revealed that the migratory and invasive cells were collectively declined in ovarian carcinoma cells transfected with si-KPNA2 (Fig. 8c and d). Finally, the expression of KIF4A was inhibited by KPNA2 knockdown of KPNA2 in ovarian carcinoma cells (Fig. 8e and f). Above data elucidated that KPNA2 could promote ovarian carcinoma cell progression by up-regulating KIF4A in vitro.

## Discussion

KPNA2 is proposed as a crucial adaptor in nucleocytoplasmic transport, which has been suggested to be a transporter of key cell cycle regulators and DNA damage reaction molecules in tumors [3, 17]. Studies have revealed that KPNA2 is abnormally expressed and as a poor prognosis predictor for multiple malignancies, including hepatocellular, bladder and breast carcinoma, and malignant melanoma [9, 16, 17, 30]. However, the prognostic value of KPNA2 expression in ovarian carcinoma is still ambiguous.

To explore the effect of KPNA2 in the development, progression, and prognosis of ovarian carcinoma, we detected extensive gene expression profiling with pre-defined parameters in ovarian carcinoma and normal samples. According to GEPIA, KPNA2 was significantly overexpressed in ovarian cancer tissues than in normal controls. Using Oncomine, we found that KPNA2 is significantly upregulated in ovarian mucinous adenocarcinoma, ovarian endometrioid adenocarcinoma, ovarian clear cell adenocarcinoma, ovarian serous adenocarcinoma, ovarian serous surface papillary carcinoma, and ovarian serous cystadenocarcinoma. Furthermore, the mutations of KPNA2 were identified by cBioPortal database. We found that mutations in ovarian cancer mainly occur in Arm domain, and the R197* and S140F mutations might have some influences on protein structure, and then may cause protein function abnormal. Prognosis analysis revealed that the higher expression of KPNA2 correlated significantly with reduced OS. We further adopt our own results validated that overexpression of KPNA2 and worse probabilities of survival in ovarian carcinoma, which determined that KPNA2 mRNA expression may be an independent prognostic biomarker in ovarian carcinoma patients.

By mining co-expression and correlation analysis data, we demonstrated that KPNA2 and KIF4A were both overexpressed in ovarian carcinoma. KIF4A, a member of the kinesin superfamily (KIFs), is associated with multiple cellular activities, particularly spindle formation and centrosome assembly in mitosis, chromosome concentration and separation [5, 27]. Furthermore, multiple studies have demonstrated that KIF4A is closely correlated with the occurrence and development of various malignancies, such as lung adenocarcinoma [34], hepatocellular carcinoma [15], colorectal cancer [14], prostate cancer [5]. Then, we identified the expression of KIF4A in ovarian carcinoma through GEPIA database and our own samples. The results determined that KIF4A mRNA expression was significantly up-regulated in ovarian carcinoma samples compared with controls. These results illustrate that the expression of KPNA2 may regulate tumor invasion and metastasis correlated with KIF4A transcription.

Then, we further validated the function of KPNA2 in growth and metastasis of ovarian carcinoma cells through knockdown the expression of KPNA2 using siRNA technology. The experiment demonstrates that KPNA2 might be a potent factor promoting the proliferation, migration, and invasion of ovarian carcinoma. Moreover, we identified the expression level of KIF4A after KPNA2 knockdown in ovarian cancer cell lines. As anticipated, we found that the down-regulation of of KIF4A expression after KPNA2 knockdown in ovarian cancer cell lines. These experiments preliminarily validate that KPNA2 exerted its effects on the malignant behaviors of ovarian cancer cells through the KIF4A signaling pathway.

## Conclusion

In general, the present study confirm that KPNA2 is highly expressed in ovarian carcinoma, as well as an independent risk factor for poor prognosis in patients with ovarian carcinoma. Also, we elucidate that KPNA2 has a role in malignancy mainly through the KIF4A signaling, which is a potential target for treating ovarian cancer. However, our understanding of the regulatory mechanism of KPNA2 in ovarian carcinoma is limited and requires further exploration.

## Data Availability

The analyzed data sets generated during the study are available from the corresponding author on reasonable request.
